# Supplementary material for: An Oxylipin-Related Nutrient Pattern and Risk of Type 1 Diabetes in the Diabetes Autoimmunity Study in the Young (DAISY)
Source: Nutrients. 2023 Feb 14;15(4):945. doi: 10.3390/nu15040945 (PMC9962656; doi:10.3390/nu15040945)
Supplement: Supplementary file 1 [file nutrients-15-00945-s001.zip › nutrients-2188613-supplementary.pdf]

Supplemental Tables and Figures

**Table S1: PCA Loadings of Average Oxylipin Levels in 343 subjects**			
Oxylipin	Precursor FA	**PC1 loading Eigenvalue:** 9.961	**PC2 loading Eigenvalue:** 4.404
9(10)-EpOME	LA	**72**	-7
9,10-DiHOME	LA	**83**	-15
9,12,13-TriHOME	LA	**33**	-13
9-HODE	LA	**84**	0
9-HOTE	LA	**70**	14
9-KODE	LA	**73**	7
12(13)-EpOME	LA	**81**	-23
12,13-DiHOME	LA	**86**	-12
13-HOTE	LA	**75**	-4
13S-HODE	LA	**85**	-10
5-HETE	ARA	0	**71**
5,6-DiHETrE	ARA	16	**60**
8,9-DiHETrE	ARA	11	**39**
8S-HETE	ARA	-3	**40**
9-HETE	ARA	4	**49**
11,12-DiHETrE	ARA	**35**	**65**
11-HETE	ARA	4	**76**
12S-HETE	ARA	8	**71**
14,15-DiHETrE	ARA	**38**	**63**
15-HETE	ARA	1	**58**
15-KETE	ARA	0	-3
Lipoxin A4	ARA	-8	-7

LTB4	ARA	4	12
PGF2alpha	ARA	3	11
Alpha-9(10)-EpODE	ALA	**79**	-2
9,10-DiHODE	ALA	**83**	-16
alpha-12(13)-EpODE	ALA	**76**	-20
12,13-DiHODE	ALA	**74**	-5
15,16-EpODE	ALA	**80**	0
15,16-DiHODE	ALA	**74**	-24
4-HDoHE	DHA	-5	7
14-HDoHE	DHA	6	**45**
17-HDoHE	DHA	8	5
19,20-DiHDPE	DHA	**42**	**37**
15-HEPE	EPA	7	10
17,18-DiHETE	EPA	**29**	**28**

Subject-specific intercepts of 36 oxylipins were used in a Principal Components Analysis (PCA). Based on scree plot, the first two principal components (PCs) were extracted. Loading values greater than ±0.277501 (the root mean square of all loading values in the PCA) are flagged (see **bold** font). For ease of presentation, loadings are multiplied by 100 and rounded to the nearest integer.

Figure S1: Scree plot from PCA of average oxylipin levels in 343 DAISY Children

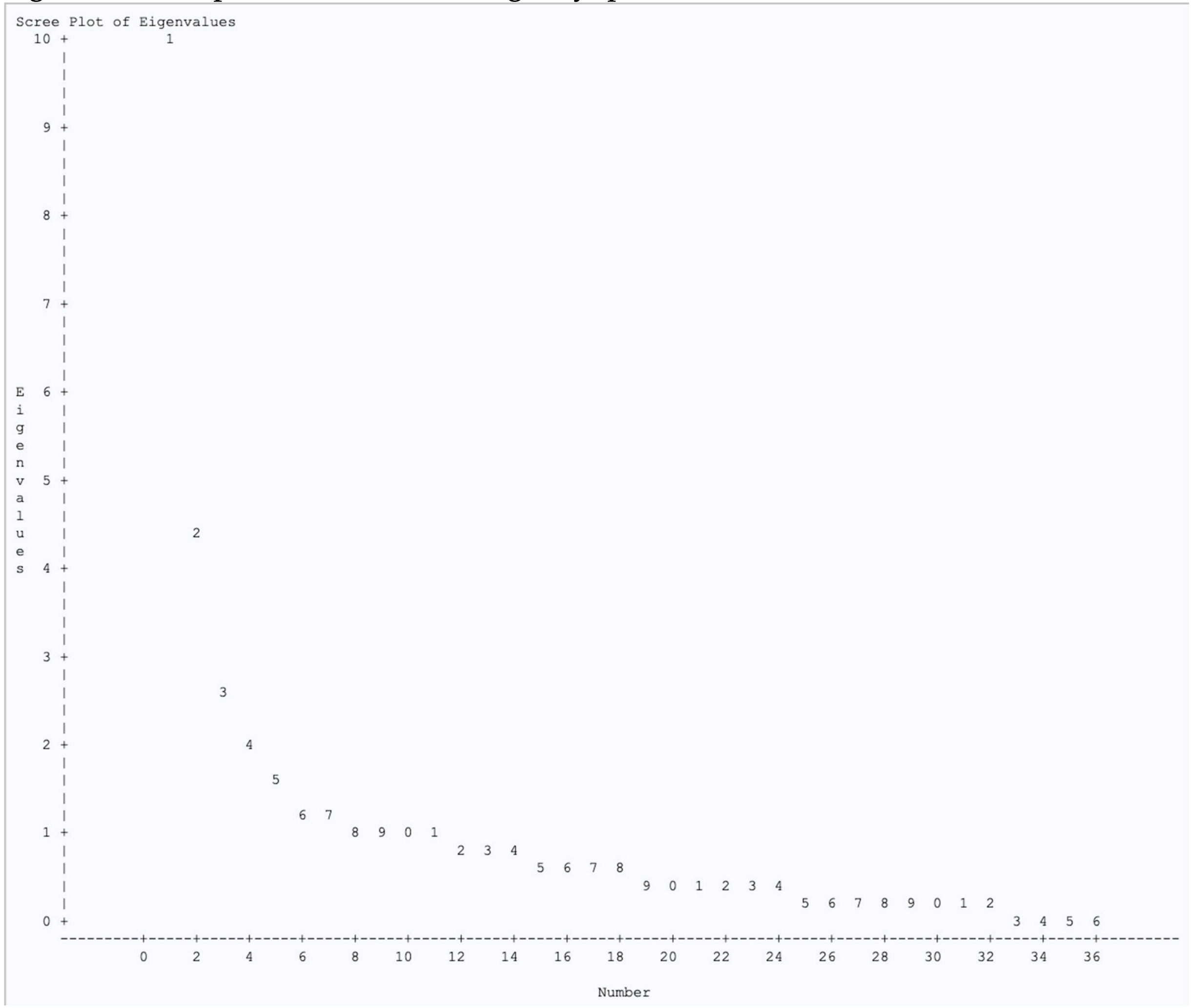

Figure S1: Scree plot of loadings from Principal Components (PCs) of subject specific intercepts of 36 oxylipins that were used in a Principal Components Analysis (PCA).

Table S2: SNPs selected through stepwise linear regression for oxylipin PC1 and PC2						
**Oxylipin PC1 Stepwise Selected SNPs**						
SNP	rs number	Gene location	β	P	MAF	Rsq
chr1:15310736:G:A	rs191841101	NRXN3: Intron Variant	0.64	2.48×10^{-5}	0.020	0.838
chr1:28663212:G:C	rs188283366	Between TAF12-DT and GMEB1	1.35	4.78×10^{-7}	0.014	0.840
chr1:237330752:G:A	rs146712883	RYR2: Intron Variant	0.91	9.68×10^{-6}	0.025	0.772
chr2:215892444:A:G	rs6720220	Between LINC00607 and LOC106373870	0.51	2.17×10^{-11}	0.178	0.899
chr6:102903894:GA:G	rs68131263	Between TARDBPP5 and LOC105377916	0.32	1.59×10^{-7}	0.289	0.993
chr7:104769392:T:A	rs11773056	LHFPL3: Intron Variant	0.29	1.63×10^{-7}	0.392	0.957
chr7:143384740:A:G	rs11773056	LHFPL3: Intron Variant	-0.28	2.17×10^{-5}	0.177	0.941
chr8:30236711:G:T	rs9942727	LOC124901928	1.23	1.96×10^{-7}	0.011	0.978
chr11:60799757:C:T	rs11230483	MS4A10: Intron Variant, LOC105369322: Intron Variant	0.23	1.74×10^{-5}	0.465	0.975
chr18:10735551:T:C	rs7238851	PIEZO2: Intron Variant	0.26	1.57×10^{-5}	0.262	0.985
chr19:3497243:C:T	rs62130574	DOHH: Intron Variant, LOC105372245: Intron Variant	0.43	9.90×10^{-8}	0.121	0.810
chr19:9675525:C:T	rs4804454	ZNF562: 2KB Upstream Variant	-0.44	4.63×10^{-6}	0.079	0.888
chr21:34932731:G:A	rs28610975	RUNX1: Intron Variant	-0.56	3.56×10^{-6}	0.067	0.894
**Oxylipin PC2 Stepwise Selected SNPs**						
chr1:200844321:G:A	rs184536095	CAMSAP2: Intron Variant	0.66	2.24×10^{-5}	0.029	0.949
chr2:214225099:A:G	rs75515363	SPAG16: Intron Variant	-0.76	5.64×10^{-7}	0.036	0.987
chr3:22312030:C:T	rs17302621	ZNF385D: Intron Variant	0.24	2.13×10^{-5}	0.443	0.981
chr6:153985398:C:T	rs62434440	Between HMGB3P19 and OPRM1	-0.47	2.75×10^{-7}	0.095	0.947
chr10:4913583:C:T	rs72769943	AKR1C6P: Intron Variant	-0.7	1.40×10^{-9}	0.059	0.940
chr12:84219134:CA:C	rs560732198	Between LOC107984536 and LOC124902976	1.11	2.67×10^{-6}	0.012	0.931
chr13:63558805:G:A	rs181948752	Between LOC124903236 and LOC105370237	1.11	7.90×10^{-9}	0.017	0.955
chr14:36769800:T:A	rs712327	SLC25A21: Intron Variant	-0.23	8.17×10^{-6}	0.389	0.944
chr15:31019385:C:A	rs12442333	TRPM1: Intron Variant	0.49	1.95×10^{-5}	0.074	0.968
chr18:13374169:G:A	rs12608222	LDLRAD4: Intron Variant	0.95	7.02×10^{-7}	0.033	0.869
chr21:32172258:T:C	rs78426270	MIS18A: Intron Variant	0.64	2.11×10^{-5}	0.025	0.886

chr22:46047091:C:G	rs111847135	LOC124905135	0.91	1.88×10^{-12}	0.035	0.898
For Oxylipin PC1, Adjusted r^2 was 0.5583. There were 13 SNPs selected (F-value: 33.81)<br>For oxylipin PC2, adjusted r^2 was 0.5588. There were 12 SNPs selected. (F value: 34.41)						

**Table S3: List of non-overlapping nutrients to test for inclusion in the reduced rank regression model**
Total Sugars
AOAC fiber
Cholesterol
Monounsaturated fat
Saturated fat
Linoleic Acid (LA)
Alpha Linolenic Acid (ALA)
Arachidonic Acid (ARA)
Eicosapentaenoic Acid (EPA)
Docosapentaenoic Acid (DPA)
Docosahexaenoic Acid (DHA)
Protein
Alpha Carotene
Beta Carotene
Beta Cryptoxanthin
Lutein and Zeaxanthin
Lycopene
Vitamin B1
Vitamin B2
Vitamin B6
Vitamin B12
Vitamin C
Vitamin D

Vitamin E
Vitamin K1
Choline
Folate
Niacin
Pantothenic acid
Calcium
Copper
Iron
Iodine
Potassium
Magnesium
Manganese
Phosphorous
Zinc
Acrylamide
Sodium
Anthocyanidins
Flavonoids
Flavan-3-ols
Flavanone
Flavones
Flavonols
Theaflavin and Polymers, Proanthocyanidins
Flavonoids, No Proanthocyanidins

Table S4: Nutrient Loadings for NP1 and NP2 derived using reduced rank regression with genetically-adjusted oxylipin profiles

Nutrient	NP1	NP2
Beta Cryptoxanthin	-0.441	-0.296
Total Flavanone	-0.360	-0.122
Vitamin C	-0.234	-0.327
Total Sugars	-0.228	-0.009
Iron	-0.216	0.100
Potassium	-0.138	-0.475
Total Flavonols	-0.073	-0.391
Magnesium	-0.039	-0.402
Vitamin B12	0.090	-0.345
Linolenic	0.196	-0.059
Lycopene	0.252	-0.013
Total Anthocyanidins	0.282	-0.094
Linoleic	0.285	-0.325
Sodium	0.475	-0.053

Figure S2: Association between NP1 nutrient components and T1D in the nested case-control study

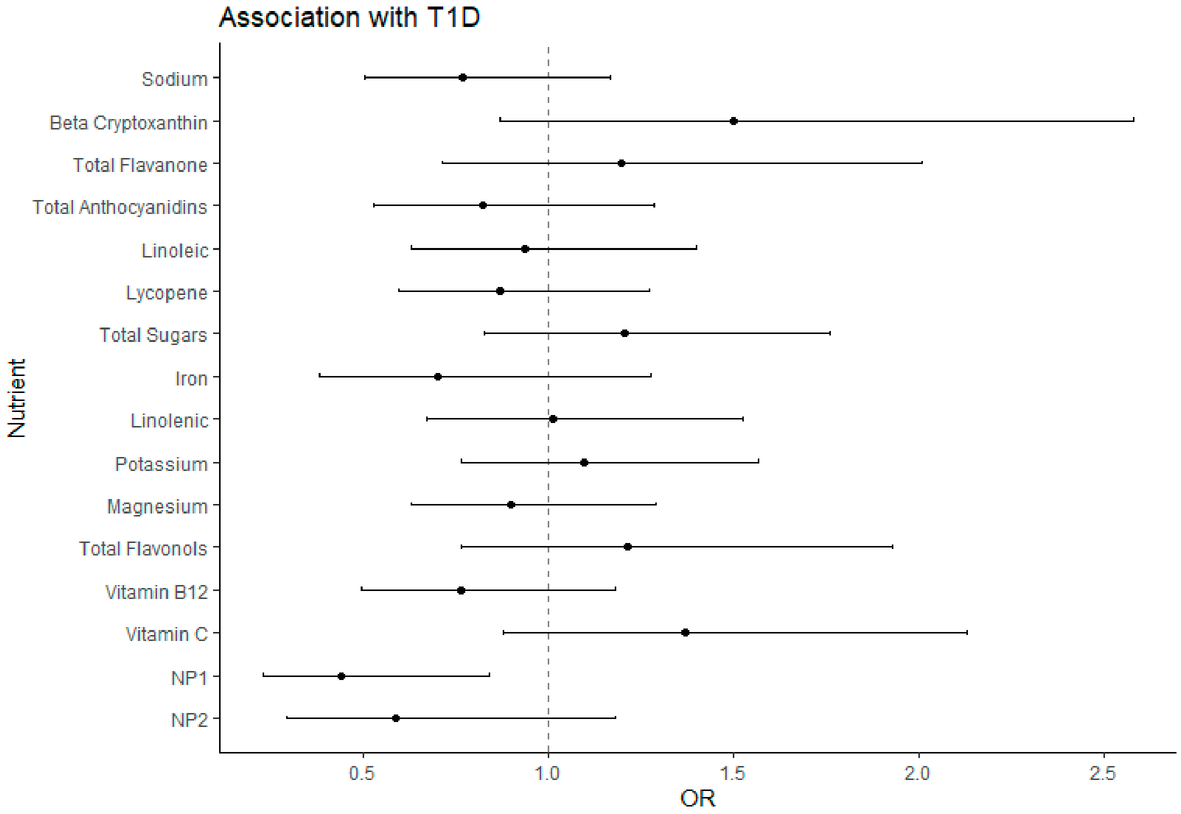

Figure S2: Logistic regression of individual nutrients that load significantly on NP1 and T1D in the T1D nested case-control study (n=69 T1D cases, 69 T1D controls), adjusted for sex, family history of T1D, race-ethnicity, and high-risk HLA genotype
